# Behavior of DNA-lacking mitochondria in *Entamoeba histolytica* revealed by organelle transplant

**DOI:** 10.1038/srep44273

**Published:** 2017-03-13

**Authors:** Makoto Kazama, Sanae Ogiwara, Takashi Makiuchi, Kazuhiro Yoshida, Kumiko Nakada-Tsukui, Tomoyoshi Nozaki, Hiroshi Tachibana

**Affiliations:** 1 Department of Infectious Diseases, Tokai University School of Medicine, Isehara, Kanagawa 259-1193, Japan; 2 Support Center for Medical Research and Education, Tokai University, Isehara, Kanagawa 259-1193, Japan; 3 Department of Parasitology, National Institute of Infectious Diseases, Tokyo 162-8640, Japan; 4 Graduate School of Life and Environmental Sciences, University of Tsukuba, Ibaraki 305-8577, Japan

## Abstract

The anaerobic protozoan parasite *Entamoeba histolytica* has mitosomes that are mitochondria lacking some canonical functions and organelle DNA. Mitosomes play an important role in the life cycle of the parasite. The distribution of proteins in mitosomes is not uniform, and how mitosomes are maintained and retained is unknown. To answer these questions, we developed a transplant method for mitosomes with hemagglutinin-tagged protein into recipient cells containing mitosomes with Myc-tagged protein. Immunofluorescence staining showed that the two protein tags colocalized in single mitosomes in some recipient cells. These results suggest that our transplant method can be used in anaerobic protozoa and that donor mitosomes may obtain recipient proteins through fusion with other mitosomes or through *de novo* synthesis of proteins in recipient cells.

The protozoan parasite *Entamoeba histolytica* is a significant cause of disease worldwide, including 50 million cases of colitis and liver abscess and up to 100,000 deaths annually^1^. *E. histolytica* has a simple life cycle, existing as either an infectious cyst or an amoeboid trophozoite that ingests bacteria and food particles and reproduces by binary fission in the host intestine. This parasite is adaptive to anaerobic conditions and has mitosomes that have largely lost canonical mitochondrial functions such as the respiratory chain and TCA cycle^2^,^3^.

Mitosomes are found in some anaerobic/microaerophilic protists and fungi^4^. The features of mitosomes shared with mitochondria include a double membrane structure^5^ and use of a mitochondrial chaperonin^6^, but mitosomes lack organelle DNA^7^. Mitosomes in *E. histolytica* play a role in the sulfate activation pathway^3^. Repression of proteins for sulfate activation^8^ and protein transport to mitosomes^9^ causes cell growth retardation, suggesting that the functions and biogenesis of mitosomes are important for proliferation of *E. histolytica*. Hence, the quality of mitosomes has to be maintained, but the quality control systems for DNA-lacking mitochondria in anaerobic microorganisms are unknown.

In mitochondria of aerobic eukaryotes, quality control includes digestion by autophagy or mitophagy^10^,^11^ and complementation of mitochondrial components, including self DNA, by mitochondrial fusion^12^,^13^,^14^,^15^. These control systems are important for prevention of mitochondrial dysfunction, incompatibilities between proteins encoded in nuclear and mitochondrial genomes, and mitochondrial diseases^16^. The complementation mechanism has been discovered by transplants of mitochondria using microinjection^12^ and cell fusion^13^.

To understand the behavior of mitosomes in *E. histolytica*, we performed transplant of mitosomes by microinjection and observed the exogenous mitosomes. In this study, we carried out transplant experiment using *E. histolytica* strains expressing mitosomal proteins with epitope tags that can discriminate between mitosomes derived from donor and recipient. The results of transplants suggest possible maintenance systems in which exogenous mitosomes associate with endogenous mitosomes or receive *de novo* synthesized proteins in the recipient. This is the first report describing transplant of DNA-lacking mitochondria into an anaerobic microorganism.

## Results

### Confirmation of AS-Myc expression in a recipient cell

To discriminate mitosomes derived from donor and recipient, we prepared an *E. histolytica* strain expressing ATP sulfurylase (AS; XP_653570, an authentic mitosomal protein)^3^ with a Myc-epitope tag at the C-terminus. Expression of AS-Myc was confirmed by western blotting and indirect immunofluorescence staining (Methods, Fig. S1). The results indicated that AS-Myc could be used to identify mitosomes in the recipient.

### Evaluation of mitosomes isolated using a modified method

**Figure 1:**
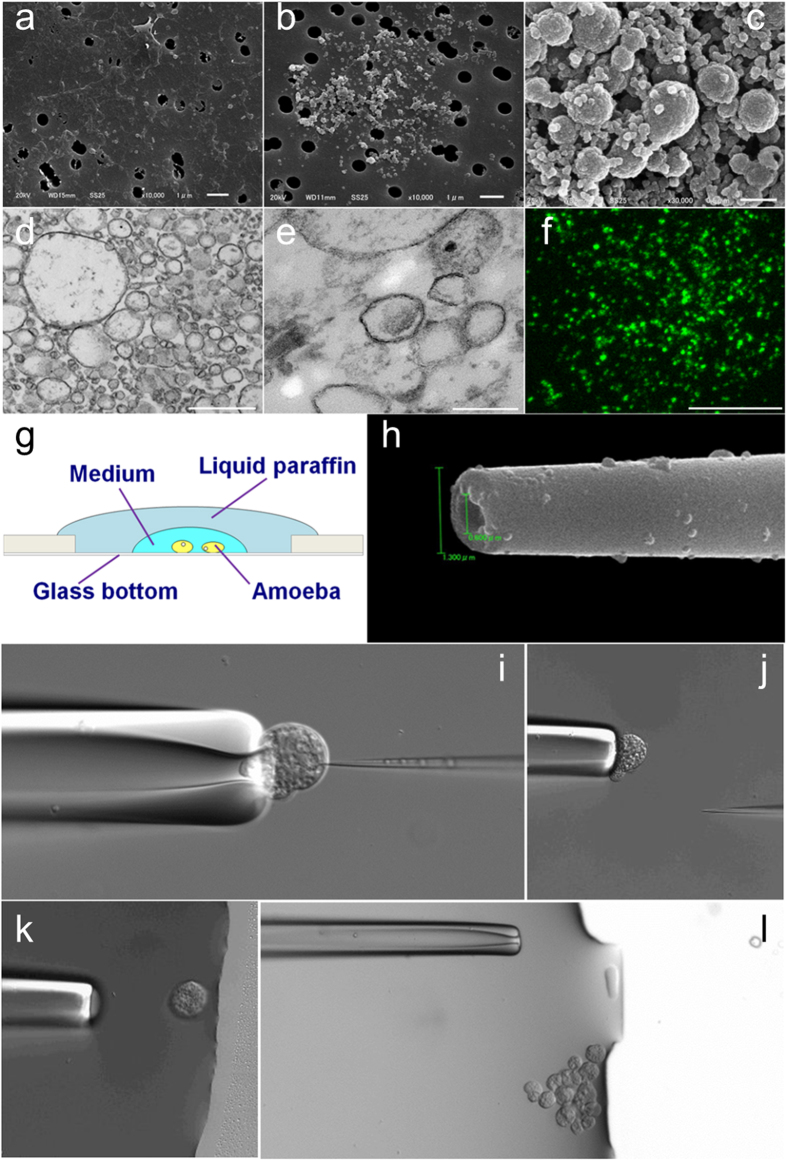
Microinjection of mitosomes into *E. histolytica* trophozoites. Mitosomes were isolated by the Percoll-PLUS gradient method. (**a**) SEM image of the supernatant of cell lysate before gradient centrifugation. (**b**) SEM image of the mitosome fraction after gradient centrifugation. (**c**) SEM image of the fraction concentrated by centrifugation. (**d–e**) TEM images of concentrated fractions. (**f**) Fluorescence image of the concentrated fraction. Mitosomes with APSK-HA were stained using anti-HA-tag-Alexa Fluor 488. (**g**) Schematic illustration of the stage on the microinjection system. (**h**) SEM image of the tip of the injection capillary. **(i–l**), Time-lapse images during transplant of mitosomes (see Video S1). Bars indicate 1 μm in (**a)** and (**b**), 500 nm in (**c**) and (**d**), 200 nm in (**e**), and 10 μm in (**f**).

**Figure 2:**
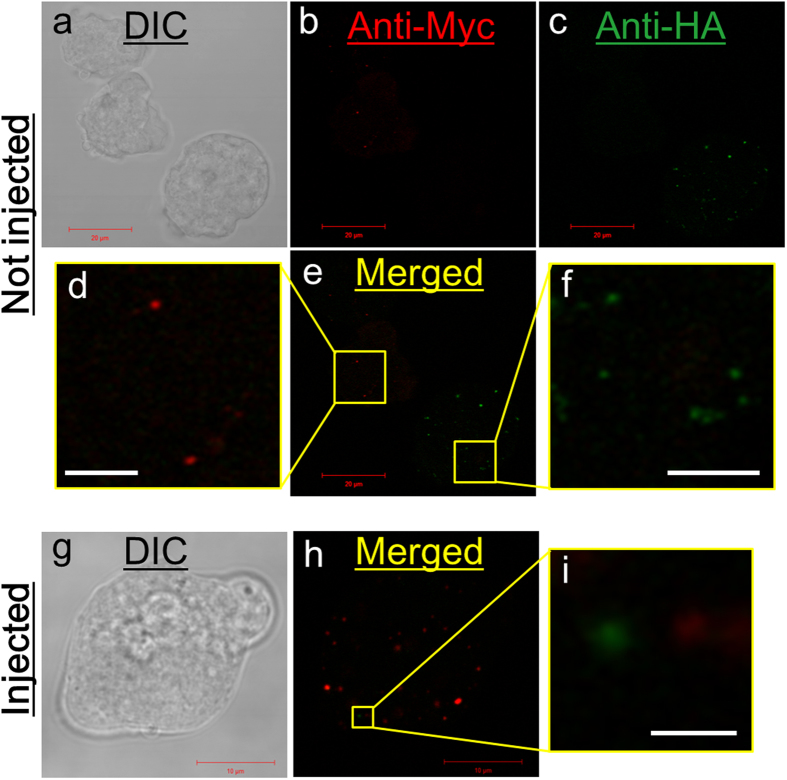
Detection of mitosomes by immunofluorescence staining in trophozoites. (**a–f)** Trophozoites expressing AS-Myc and APSK-HA were co-cultured and stained by immunofluorescence with anti-Myc-Alexa594 and anti-HA-Alexa488 antibodies. (**a**) DIC image. (**b,c**) Fluorescence images of Alexa594 and Alexa488, respectively. (**e**) Merged image of (**b**) and (**c)**. (**d, f)** Magnified images of the boxes in (**e**). (**g–i)** Trophozoite expressing AS-Myc after transplant with mitosomes containing APSH-HA. (**g**) DIC image. (**h**) Immunofluorescent image with anti-Myc-Alexa594 and anti-HA-Alexa488 antibodies. **(i)** Magnification of the box in (**h**). Bars indicate 20 µm in (**a**)-(**c**) and (**e**), 10 µm in (**g**) and (**h**), 5 µm in (**d**) and (**f**), and 1 µm in (**i**).

An *E. histolytica* strain expressing adenosine-5′-phosphosulfate kinase (APSK; XP_656278, a marker protein for *Entamoeba* mitosomes^3^) with a hemagglutinin (HA)-epitope tag (APSK-HA) was used to identify donor mitosomes for transplant. The mitosome fraction was obtained from cell lysate by the Percoll-gradient method^3^ with some modifications (see Methods). The main change was substitution of Percoll with Percoll-PLUS in gradient formation because Percoll showed lethal toxicity in cells after injection in preliminary experiments. In each fraction, the presence of Cpn60, a specific mitosome protein, was confirmed by Western blotting (Fig. S2). A scanning electron microscope (SEM) image of the contents of cell lysate before Percoll-PLUS gradient centrifugation is shown in Fig. 1a and images of the fraction after centrifugation are shown in Fig. 1b–f. Globular organelles of 0.2–0.7 μm diameter were concentrated in the fraction after centrifugation. The fraction was washed and concentrated by centrifugation (Fig. 1c). Transmission electron microscope (TEM) images (Fig. 1d,e) showed bilayer membrane structure, and direct immunofluorescence staining with anti-HA antibody also indicated spotted signals (Fig. 1f). The main diameter of these signals was <1 μm, similar to those in trophozoites (Fig. 2). These organelles were identified as mitosomes based on size, morphology, immunofluorescence staining and Cpn60 content in Western blotting. The concentration of the organelle was estimated to be 13.7 ± 3.8 per picoliter based on immunofluorescence images.

### Transplant with mitosomes

Using an injection capillary with a 1.3–2.0 μm outer diameter and a 0.6–1.3 μm inner diameter on the tip (Fig. 1h), the transplant process (Fig. 1i–l) was performed by microinjection (see Methods and Video S1). The injected volume was estimated to be approximately 0.29–0.75 picoliter (see Supplemental information and Fig. S3). Therefore, the number of incorporated mitosomes was estimated to be 2.8–13 per recipient cell.

Just after injection, the recipient cell moved or a part of the cell became swollen on the tip of the holding pipette. Vomiting behavior of injected cells was not observed. Injected cells were carried to a restricted area on the dish (Fig. 1l) and some were transferred to another dish filled with fresh medium. After sealing, these cells were incubated at 37 °C and traced by tiling and time-lapse images taken at 30-min intervals (Video S2). The injected cells showed amebic motion and divided at about 20 h after injection. The daughter cells underwent the next division asynchronously at about 29 h and 34 h after injection. *Entamoeba* trophozoites sometimes require the assistance of a neighboring cell that mechanically severs the tubular bridge between two daughter cells^17^,^18^, but this midwife cell behavior was not observed in these divisions. The injected cells and their daughter cells showed regular binary fission. These results indicate that the injected cells are viable and retain the ability to proliferate.

### Detection of donor mitosomes in recipient cells

Signals for APSK-HA were detected in recipient cells from three strains (Table 1) using direct immunofluorescence staining with anti-HA antibody. The diameter of the fluorescence signals ranged from 0.6 to 1.0 μm, the same size as that for signals in donor cells (Fig. S4). Injected cells with donor mitosomes were observed in approximately 23.5% of examined cells from strain HM-1:IMSS within 30 min after microinjection. The number of donor mitosomes in the recipient cell was scored as 0 to 15 per cell using this strain, and the density was estimated to be 0.88 donor mitosomes per examined cell. In Table 1, the transplant efficiency is expressed as the rate of cells with donor mitosomes per number of examined cells. These values were equal to or greater than those for transplant of mitochondria^12^ or exogenous cytoplasm^19^ in previous studies. The detection of donor mitosomes in recipient cells after microinjection indicates that our transplant method for mitosomes was successful.

### Time-dependent decrease of donor mitosomes

We used a combination of APSK-HA and AS-Myc to identify donor and recipient mitosomes, respectively, in recipient cells. Immunofluorescence images of this mixture with strains expressing AS-Myc and APSK-HA are shown in Fig. 2a–f. Fluorescence signals were detected independently in each specimen by double staining. Each cell showed a single signal alone and these signals did not overlap. After injection with mitosomes from the strain expressing APSK-HA, the injected cell showed several signals for AS-Myc and a few signals for APSK-HA in a single layer on the Z-axis (Fig. 2g–i). When trophozoites expressing AS-Myc were used as recipient cells, the transplant efficiency (24.8 ± 18.1%, mean ± SD) was the same as that with strain HM-1:IMSS (27.1 ± 22.5%, *p* = 0.862). However, the transplant efficiency decreased with post-incubation time to 8.7 ± 12.2% and 1.3 ± 3.1% at 60 and 120 min after microinjection, respectively (the time includes the microinjection step). The efficiency differed significantly between 30 and 60 min (*p* = 0.04) and between 30 and 120 min (*p* = 0.003).

### Staining patterns of mitosomes in recipient cells expressing AS-Myc

**Figure 3:**
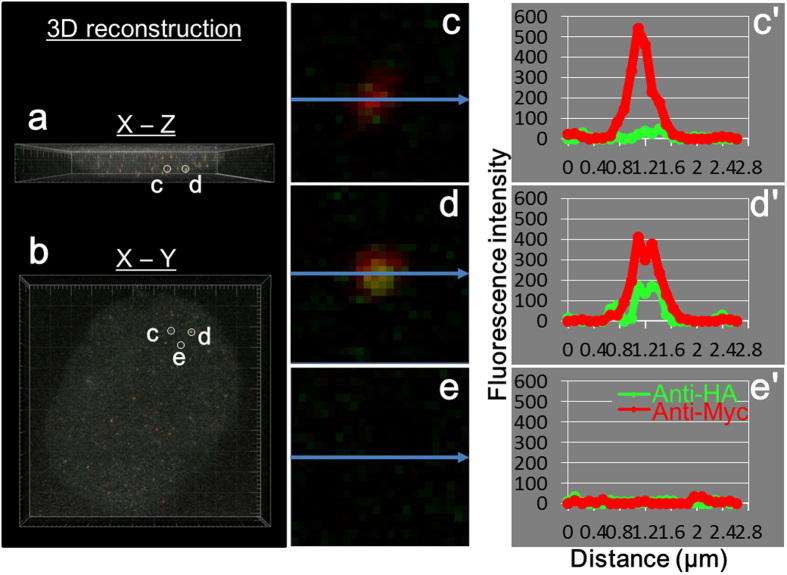
Three-dimensional reconstruction of an injected cell and analysis of fluorescence intensity. Z-stacking images were reconstructed by Imaris software. (**a**) X-Z and (**b**) X-Y images were shown and a rotated movie file (Video S3) was obtained from the same data. (**c–e)** Enlarged view of fluorescent images from (**b**). (**c′–e′**) Intensities correspond to (**c–e)** and are shown along arrows. Areas (**c)** and (**d**) contain mitosome signals. Background signals are collected in area (**e**).

Recipient cells expressing AS-Myc after transplant of mitosomes containing APSK-HA were analyzed three-dimensionally (Fig. 3a and b, and Video S3). Donor and recipient signals were scattered in the cells. Figure 3 showed a recipient cell in the X-Z (Fig. 3a) and X-Y axis view (Fig. 3b). Figure 3c–e showed in an enlarged view, and the corresponding fluorescence intensities were Fig. 3c′–e′. In Fig. 3c, Myc-signal showed high fluorescence intensity and HA-signal was very low, similar to background noise (Fig. 3e). The image and intensities in Fig. 3c and c′ indicate that the mitosome contained AS-Myc, but not APSK-HA. In contrast, overlapping fluorescence signals for AS-Myc and APSK-HA were observed in a single mitosome (Fig. 3d) and both intensities were higher than the background level (Fig. 3d′ and e′).

**Figure 4:**
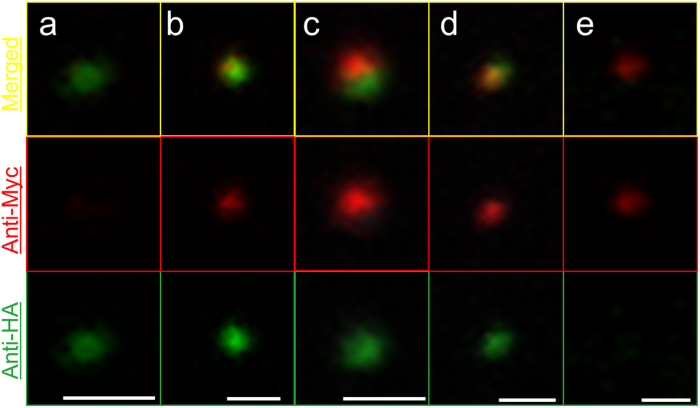
Fluorescence patterns of mitosomes. Mitosomes from different injected cells are shown in each channel and as a merged view. Bars indicate 1 μm.

Various staining patterns of mitosomes are shown in Fig. 4. The mitosomes in Fig. 4a and e show a single fluorescent signal of AS-Myc and APSK-HA, whereas Fig. 4b–d show colocalization of two signals with different merged patterns. The two fluorescent signals in Fig. 4b and c are close, but do not overlap completely, and the green area is larger than the red area in both mitosomes. In contrast, the image in Fig. 4d shows an overlapped yellow region. Four and nine merged signals were detected from 76 cells within 30 min and from 58 cells within 60 min after injection (Table 1). Given the number of incorporated mitosomes in each cell (2.8–13), the rates of merged signals per total injected mitosomes was calculated as 0.4–1.9% and 1.1–5.5% within 30 min and 60 min after injection, respectively.

**Figure 5:**
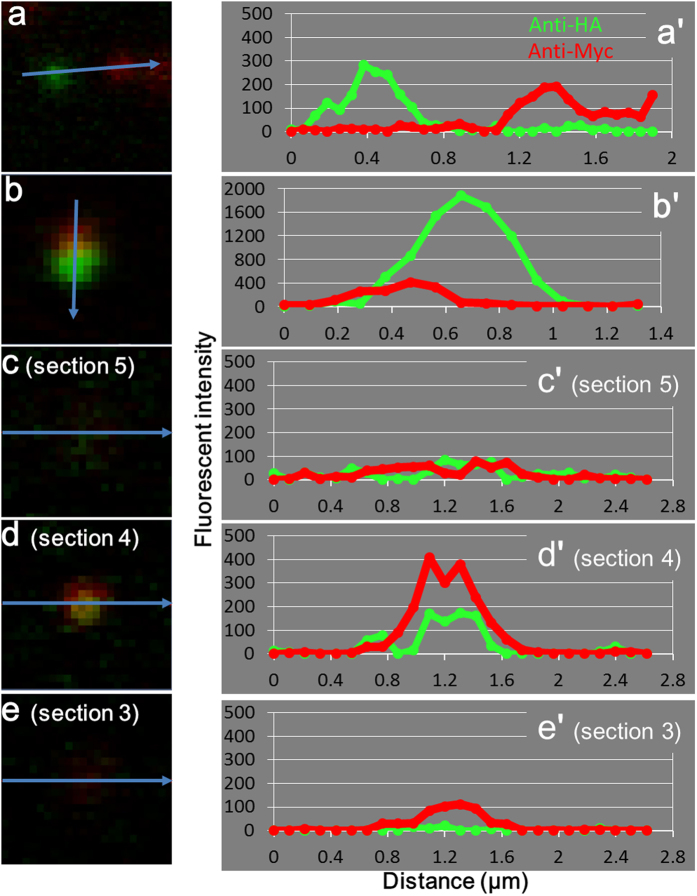
Analysis of fluorescent intensities in mitosomes. Fluorescent intensities in mitosomes are shown along arrows. (**a–e)** Images of mitosomes. (**a′–e′**) Intensities corresponding to (**a–e).** Image (**a**) obtained from Fig. 2i. Images (**c–e)** were three continuous sections in 11 Z-stacking images of the single mitosome in Fig. 3d. Z-stacking images were taken by laser microscopy with 1.1-μm thickness sections and a 0.53-μm merge.

Analysis of fluorescence intensity showed three types of localization patterns of the two signals. In Fig. 5a, two mitosomes are shown with each signal independently localized in the recipient cell. This pattern of a single color was also shown in Fig. 2 and for mitosomes in Fig. 4a and e. In the pattern in Figs 4b,4c and 5b, fluorescence signals co-localize in single mitosomes, with the peaks of intensity from APSK-HA (donor) and AS-Myc (recipient) signals close together and partially overlapped. A single mitosome on three continuous Z-stack sections with an identical position on the X-Y axis is shown in Fig. 5c–e. This mitosome is identical to that in Fig. 3d and shows completely overlapped peaks on Z-stacking. These staining patterns were sometimes observed in a cell, such as for the mitosome in Fig. 4d. Colocalization of both fluorescence peaks demonstrates that proteins from donor and recipient were expressed in the same mitosome. These observations suggest that single mitosomes in transplanted cells contained APSK-HA and AS-Myc.

### Comparison of transplant efficiency with HM-1:IMSS and G3

The transplant efficiency in strain G3 (57.6 ± 23.9%) was higher than that in HM-1:IMSS (27.1 ± 22.5%) within 30 min after microinjection, although the difference was not significant (*p* > 0.05). Strain G3 is a genetically modified avirulent strain from the virulent *E. histolytica* strain HM-1:IMSS^20^,^21^. Expression of mitosomal proteins occurs at similar levels in strains G3 and HM-1:IMSS based on transcript expression array profiles in AmoebaDB (http://amoebadb.org/amoeba, summarized in Table S1). Genes with different expression levels in these strains are listed in Tables S2 and S3. For example, *AIG1* (avirulence inducing gene 1), which is similar to plant *AIG* genes involved in bacterial resistance^22^, is strongly expressed in strain G3 (Table S2). Amoebapore, which was silenced in establishment of strain G3^20^,^21^, is markedly downregulated (Table S3).

## Discussion

This study has four key findings: (1) microinjection can be used for transplant of mitosomes in the anaerobic protozoa, *E. histolytica*, (2) mitosomes derived from donor and recipient can be recognized in single cells using labeling with two epitope tags, (3) transplant efficiency decreases with time after microinjection, and (4) donor mitosomes in recipient cell show alternative characteristics with both proteins of donor and recipient.

Transplant of mitosomes by microinjection was applicable to cells in *E. histolytica* with high efficacy. Using the microinjection method, Flickinger *et al*.^19^ performed successful transplant of cytoplasm including mitochondria between free-living amoebas *Amoeba discoides* and *Pelomyxa carolinensis.* In our transplant experiments, we used an established method to isolate mitosomes^3^ that were of size 1–2 μm on fluorescence microscopy^6^ and were estimated to be <500 nm on high-resolution laser scanning sections^7^. The small organelles could pass through capillaries with similar diameters and are easily handled using a single recipient cell. In contrast to microinjection, cell fusion may transmit various contents of a donor cell to a transplant cell, and this method has not been established in *Entamoeba*. Therefore, microinjection is the best choice for transplant of organelles into *Entamoeba* species. As results shown in Table 1, all kinds of recipient cell using in this study showed more than 20% of transplant efficacy.

In addition, the combination with transfection techniques made it possible to identify the mitosomes by immunofluorescence staining to the expressed epitope-tags in marker proteins of *E. histolytica*. Although transplanted mitochondria are recognized by using polymorphism of mitochondrial DNA in many studies^12^,^13^,^23^,^24^,^25^, exogenous organelles could be detected without DNA probes in this method. Therefore, this is the first report of transplant with DNA-lacking mitochondria. This is also the first implementation of microinjection for the purpose of organelle transplant in anaerobic protozoa.

On the other hand, more than 40% of injected cells from all used strains showed no fluorescent signal from the donor within 30 min post-microinjection. Additionally, recipient cells derived from the strain expressing AS-Myc had reduced transplant efficiency with time, with a significant difference between 30 and 120 min after microinjection. In free-living amoebas *Amoeba discoides* and *Pelomyxa carolinensis*, which have mitochondria, recipient cells injected with exogenous cytoplasm expel the injected cytoplasm within 30 min post-injection, or exogenous organelles are segregated and digested by lysosomal enzymes in vacuole-like spaces between 6 h and 2 days post-injection^19^. Vomiting behaviors were not observed in any strains of *E. histolytica* in this study. There is a possibility that mitosomes from donors are digested by autophagosomes or lysosomes. Autophagy or mitophagy digests both damaged mitochondria^10^,^11^ and exogenous mitochondria derived from spermatozoa during fertilization in *Caenorhabditis elegans*^26^,^27^. Although the digestion mechanism of mitosomes is unknown in *E. histolytica*, we cannot exclude the possibility that mitosomes were digested by autophagy or mitophagy, similarly to mitochondria. Transplant with a larger number of donor mitosomes would allow observation of organelle degradation in *E. histolytica*.

Cells derived from strain G3 showed the highest efficacy among cells derived from all strains, although there were no significant differences among the cells (*p* > 0.05). A high fold change in strains HM-1:IMSS and G3 was found for AIG1 in transcript expression array profiles in AmoebaDB AIG family proteins are classified as stress response proteins that show high levels in comparative transcriptomic analysis between virulent and avirulent strains^28^. Thus, stress responses in *E. histolytica* might be involved in the success of transplant.

The most interesting observation in this study was the overlap of signals for donor and recipient proteins in single mitosomes in injected AS-Myc expressing cells (Figs 3, 4 and 5). This observation may give a clue to resolution of the question of how mitosomes are maintained and retained in *E. histolytica* because donor and recipient were derived from an identical strain. The present result shows that some donor mitosomes contain proteins from both the donor and recipient, which suggests that mitosomal proteins are compatible in donor and recipient cells. There are two possible explanations for this phenomenon. First, AS-Myc synthesized *de novo* in the recipient cell may be transported into donor mitosomes. We also cannot exclude the possibility that APSK-HA from broken or degraded injected mitosomes is imported into recipient mitosomes. However, this is unlikely because of the estimated small number of incorporated mitosomes of 2.8–13 per recipient cell. Second, complementation of proteins between donor and recipient mitosomes may occur by fusion within the recipient cell. Two types of mitochondrial fusion are known: complete fusion with sharing of mitochondrial DNA and detachable fusion, which is referred to as “kiss-and-run” fusion^15^,^29^. The overlap of signals in mitosomes (Figs 3d and 5c–e) and partially merged signals (Figs 4c and 5b) may represent the former and latter behavior, respectively. If mitosomes fuse to each other, the significance of fusion may be to exchange components between mitosomes because the distributions of several mitosome proteins, including AS and APSK, are not uniform based on immunostaining methods, which suggests heterogeneity of mitosomes in *E. histolytica*^3^,^8^. The quantity of these proteins in mitosomes may be a rate-limiting factor in the sulfate activation pathway, which is important for proliferation of *E. histolytica*^8^. Further analyses are required to examine this possibility, but fusion may contribute to maintenance of mitosome function through complementation.

Regardless of the mechanism, donor mitosomes must be acceptable to recipient proteins, which suggests that components are added to the donor mitosome. Moreover, the acceptance of mitosomal proteins from the recipient would lead to loss of identity of the donor mitosome. Therefore, the true number of transplanted mitosomes may be higher than the number determined in the study. This possibility does not negate the result that donor mitosomes decrease time-dependently in transplanted cells. Furthermore, exogenous mitosomes in the recipient cell are maintained by association with other mitosomes and/or by receiving mitosomal proteins synthesized *de novo*. Further development of the method used in this study to transplant DNA-lacking mitochondria will produce a primary tool to evaluate the behaviors of specialized organelles in anaerobic microorganisms.

## Methods

### Cell culture

#### Trophozoites of four strains

HM-1:IMSS^30^, strains expressing APSK-HA^3^ or AS-Myc, and G3^20^,^21^ in *Entamoeba histolytica* were used in this study. These trophozoites were cultured axenically at 37 °C in BI-S-33 medium^31^ (donor) or YIMDHA-S medium^32^ (recipient) supplemented with 12% adult bovine serum. Medium for strains expressing APSK-HA and AS-Myc was supplemented with 20 μg/ml G418.

### cDNA preparation

Total RNA was isolated from trophozoites of HM-1:IMSS clone 6 with Trizol^®^ reagent (Life Technologies) and mRNA was purified using a GenEluteTM mRNA Miniprep Kit (Sigma, St. Louis, MO, USA). cDNA was synthesized from mRNA using SuperScript^TM^ III RNase H reverse transcriptase (Life Technologies) and oligo(dT)_20_ primer.

### Plasmid construction

To generate an expression plasmid (pEhEx/AS-Myc) for *E. histolytica* AS tagged with Myc at the C-terminus, *E. histolytica AS* gene was PCR-amplified from cDNA using Phusion DNA polymerase (New England Biolabs) and specific primers (sense: 5′-GTC GGG ATC CAT GAG CAT TCA AGA AAA CTT AAA C-3′ and antisense: 5′-GTC GGG ATC CTT TCA TGG CAT CAC CAG TAG C-3′ (underlined bases indicate the *Bam*H I restriction site). After *Bam*H I digestion, the digested fragment was ligated with pEhEx/Myc^33^ digested by *Bgl* II using a Ligation-Convenience Kit (Nippongene, Tokyo, Japan).

### Amoeba transformation

Transfection of trophozoites and selection and maintenance of transformants were performed based on the method of Nozaki *et al*^34^. Briefly, liposomes containing pEhEx/AS-Myc were made with 5 μg plasmid, Lipofectamine^®^ and Plus^TM^ reagent (both Life Technologies). The liposome was mixed into OPTI-MEM I medium (Life Technologies) with 5 mg/ml L-cysteine (Sigma) and 1 mg/ml ascorbic acid (Wako, Osaka, Japan). Approximately 5.0 × 10^5^ trophozoites were incubated with the medium containing the liposome at 37 °C for 5 h. After incubation, trophozoites were transferred into culture test tubes with warmed YIMDHA-S medium supplemented with 15% adult bovine serum (Sigma). Drug selection by G418 was begun from 24 h later and medium was replaced by fresh medium with G418 every 24 h.

### Mitosome isolation

Mitosomes were isolated by Percoll-gradient fractionation^3^ with modifications. Briefly, approximately 0.5–1.0 × 10^8^ trophozoites were washed three times with PBS containing 2% glucose. After resuspending in SM buffer (10 mM MOPS-KOH/250 mM sucrose, pH 7.2) containing protease inhibitor cocktail (cOmplete Tablets, Mini EDTA-free, EASYpack, Roche, Mannheim, Germany) and 50 μM E-64 (Sigma), trophozoites were disrupted mechanically with a Dounce homogenizer. After centrifugation at 5,000 × *g* for 10 min at 4 °C, the supernatant was ultracentrifuged (Optima MAX-XP Ultracentrifuge, Beckman Coulter, Brea, CA, USA) on a gradient with Percoll-PLUS (GE Healthcare Bio-Sciences AB, Uppsala, Sweden). A total of 47–48 fractions (100 μl each) were collected from top to bottom in the tube. Two fractions with OD higher than 280 nm were mixed and washed three times with SM buffer without protease inhibitors by centrifugal filter units (Amicon Ultra-0.5 mL Ultacel-100K, Merck Millipore, Tullagreen, Carrigtwohill, Ireland). The fractions were diluted to an OD_280_ range of 1.5 to 2.5 with SM buffer without protease inhibitors, stored at 4 °C, and used within 8 h after preparation.

### Microinjection devices

Glass thin-capillary tubes (GD-1 with filaments; Narishige Co., Tokyo, Japan) pulled on a P-97/IVF micropipette puller (Sutter Instrument Co., Novato, CA, USA) were used for transplant. After pulling, the capillary was bent at a 30° angle at 1.0–1.2 mm from the tip by a MF-1 micro-forge (Technical Products International, St. Louis, MO, USA). The injection capillary and a holding pipette were connected to an IM 300 Microinjector (Narishige) and hydraulic micromanipulator (NT-8, Narishige), respectively. The tip of the capillary and cells were visible on an inverted microscope (Observer Z1, Zeiss Co., Germany). The stage was kept at room temperature during microinjection. After injection, the stage was exchanged for a mechanical stage (ThermoPlate, MATS-555AXK and MATS-555RN, Tokai Hit Co., Shizuoka, Japan) to incubate cells at 37 °C. The whole injection device was placed on a vibration isolator system (ST-X, Showa Science Co., Tokyo, Japan).

### Transplant by microinjection

The injection capillary was loaded with a mitosome fraction and stored at 4 °C before connection to the microinjection system. Recipient cells for transplant were cultured in YIMDHA-S medium^18^ at 37 °C and an aliquot was dropped onto a 50-mm glass bottom dish (MatTek Corp., Ashland, MA, USA) just before microinjection. To maintain anaerobic conditions and prevent drying, the medium on the dish was overlaid with liquid paraffin (Nacalai Tesque Inc., Kyoto, Japan) (Fig. 1g).

A single recipient cell was removed from the bottom by pushing with the holding pipette. Adhered recipient cells on the bottom of the dish became spherical by removing and holding. The removed cell was held on the tip of the pipette and injected with the mitosome fraction through the injection capillary for 2 s per injection with 9.0–11.0 psi pressure of nitrogen gas. The capillary for microinjection was pulled and shaped to a 1.3–2.0 μm outer diameter and a 0.6–1.3 μm inner diameter on the tip (Fig. 1h).

After injection, the cells were carried to a restricted area on the dish (Fig. 1l), and then released from the holding pipette. Microinjections were finished within 30 min in each trial, and 5 to 20 injected cells were collected from every trial. Recipient cells during microinjection were recorded by AxioVision 4.8 software (Zeiss), as shown in Fig. S1. Some injected cells were transferred to a 35-mm glass base dish (AGC Techno Glass Co., Tokyo, Japan) filled with medium. The dish was sealed with grease applied to the cover glass and incubated at 37 °C. The remaining cells were incubated in fresh YIMDHA-S medium at 37 °C and then fixed with 4% PFA within 2 h after microinjection.

### Direct immunofluorescence staining

Cells and mitosomes after isolation were fixed with 4% PFA for 15 min and adhered to silane-coated glass slides (Muto Pure Chemicals Co., Tokyo, Japan) using a Shandon Cytospin 3 Centrifuge (Thermo Fisher Scientific, Waltham, MA, USA) at 800 rpm for 10 min. After washing with PBS for 15 min, specimens were immersed in blocking solution (1% BSA, 0.2% saponin in PBS) for 30 min at room, and then reacted with anti-HA-tag-Alexa Fluor 488 (M180-A48, MBL Co., Nagoya, Japan; 1/500 dilution in blocking solution) and/or anti-Myc-tag-Alexa Fluor 594 (M047-A59, MBL Co.; 1/200 dilution in blocking solution) at 4 °C overnight. After washing with 0.1% BSA in PBS for 15 min, glass slides were embedded with 10% glycerol in PBS containing 1.25 mg/ml DABCO and stored at 4 °C in the dark.

### Electron microscopy

Mitosomes after isolation were subjected to electron microscopy. Mitosome fractions were pre-fixed with 2.5% glutaraldehyde in 0.05 M phosphate buffer (pH 7.6) for 1 h. For SEM, specimens were transferred to a sample folder (SEMpore. JEOL, Tokyo, Japan) and then post-fixed with 1% osmium tetroxide and dehydrated in a graded ethanol series (50, 70 and 90% ethanol, and anhydrous ethanol three times). The ethanol was substituted with *t*-butanol and the sample was freeze-dried using JFD-310 (JEOL). Specimens were observed at 15 kV accelerating voltage on a JSM-6510LV (JEOL) after osmium evaporation (Neoc-Pro, Meiwafosis Co., Tokyo, Japan). For TEM, specimens were adhered to a glass slide using a Shandon Cytospin 3 Centrifuge at 800 rpm for 10 min. After post-fixation and dehydration as for SEM specimens, samples were embedded in epoxy resin (Quttol-812, Nisshin EM Corp., Tokyo, Japan). The thin-sectioned specimens were observed at 80 kV accelerating voltage using a JEM1400 (JEOL) after staining with uranyl acetate and lead citrate.

### Laser scanning microscopy

For visualization of mitosomes in cells after direct immunofluorescence staining, images were acquired in lambda (emission fingerprinting) and Z-stacking mode on a LSM 510 Meta confocal microscope equipped with a 488 nm Ar laser and a 543 nm He/He laser (Zeiss). Acquisitions were carried out at 499–745 nm using a C-Appochromat 63×/1.2 W corr. objective. Images were analyzed using LSM 510 Meta software (ZEN 2009 ver. 6.0.0.303, Zeiss). For three-dimensional analysis of cells, optical 1.1-μm sections with a 0.53-μm merge were reconstructed using Imaris software ver. 6.2.1. (Bitplane AG, Zurich, Switzerland).

### Statistical analysis

The significance of differences was evaluated by Welch *t*-test, with *p* < 0.05 considered significant and *p* < 0.01 considered highly significant.

## Additional Information

**How to cite this article:** Kazama, M. *et al*. Behavior of DNA-lacking mitochondria in *Entamoeba histolytica* revealed by organelle transplant. *Sci. Rep.* **7**, 44273; doi: 10.1038/srep44273 (2017).

**Publisher's note:** Springer Nature remains neutral with regard to jurisdictional claims in published maps and institutional affiliations.

## Supplementary Material

Supplementary Information

Supplementary Video S1

Supplementary Video S2

Supplementary Video S3

## Figures and Tables

**Table 1:** Efficiency of transplant with mitosomes containing APSK-HA into recipient strains.

Recipient	Time*	N^#^	Cells							Mitosomes		
			No. of Injected	No. of Examined	No. of Detected	Total Rate	Mean	SD	SE	No. of Detected (merged)	Max. No. per cell	Density^$^
HM-1: IMSS	30 min	4	120	34	8	0.24	0.27	0.23	0.11	30	15	0.88
AS-Myc	30 min	10	371	76	17	0.22	0.25	0.18	0.06	22 (4)	5	0.29
	60 min	8	313	58	10	0.17	0.09	0.12	0.04	32 (9)	11	0.55
	120 min	6	247	67	1	0.02	0.01	0.03	0.01	1 (0)	1	0.02
G3	30 min	3	121	24	13	0.54	0.58	0.24	0.14	42	6	1.75

Transplant efficiency is shown as the percentage of cells with donor mitosomes per number of examined cells. The number of incorporated organelles was estimated to be 2.8-13 per cell.

*Time indicates the processing time for microinjection and post-incubation until fixation.

^#^No. of Exp. (number of experiments) indicates the number of independent mitosome isolations.

^$^Number of detected mitosomes per number of examined cells.
