# Supplementary material for: Behavior of DNA-lacking mitochondria in Entamoeba histolytica revealed by organelle transplant
Source: Sci Rep. 2017 Mar 13;7:44273. doi: 10.1038/srep44273 (PMC5347163; doi:10.1038/srep44273)
Supplement: Supplementary Information [file srep44273-s1.pdf]

Behavior of DNA-lacking mitochondria in *Entamoeba histolytica* revealed by organelle transplant

Makoto Kazama[1]*, Sanae Ogiwara[2], Takashi Makiuchi[1], Kazuhiro Yoshida[1], Kumiko Nakada-Tsukui[3], Tomoyoshi Nozaki[3,4], Hiroshi Tachibana[1]*

[1] Department of Infectious Diseases, Tokai University School of Medicine, Isehara, Kanagawa 259-1193, Japan. [2]Support Center for Medical Research and Education, Tokai University, Isehara, Kanagawa 259-1193, Japan. [3]Department of Parasitology, National Institute of Infectious Diseases, Tokyo 162-8640, Japan. [4]Graduate School of Life and Environmental Sciences, University of Tsukuba, Ibaraki 305-8577, Japan

Supplementary information and data

Microinjection quantity

The microinjection volume into trophozoites of *E. invaden*s was estimated based on vacuole-like structures with different cytosol transparency that appeared just after microinjection (Fig. S3). Since the diameter of the vacuole was 6-12 μm, its volume was estimated to be 0.28 to 0.75 picoliters. Such vacuoles were not observed in *E. histolytica*. The concentration of organelles was estimated to be 13.7±3.8 organelles per picoliter, giving an estimate of approximately 2.8 to 13 organelles incorporated into a recipient cell.

Supplementary Materials

Three tables, four figures and three video files are provided with this manuscript.

Table S1. Comparison of expression levels of mitosome proteins between G3 and HM-1:IMSS strains.

Gene ID	Product Description	Fold Change (G3/HM-1:IMSS)
EHI_188880	Cpn10	1.1
EHI_101120	Hsp70	-1.1
EHI_197160	AS	-1.6
EHI_179080	APSK	-1.3
EHI_124880	IPP	-2.1
EHI_095150	AAC	-1
EHI_177580	NaS	-1.9
EHI_104420	Tom40	1.3
EHI_078220	Sam50	-2.8
EHI_053160	Tom60	-1.3
EHI_178630	MBOMP30	-1.1

Expression levels are compared for major mitosome proteins collected from *E. histolytica* G3 and HM-1:IMSS Transcript Expression Array Profiles in AmoebaDB (http://amoebadb.org/amoeba/).

Table S2. Top ten upregulated genes in strain G3 compared with strain HM-1:IMSS.

Gene ID	Product Description	Cat.	Fold Change (G3/HM-1:IMSS)
EHI_011270	Hypothetical protein, conserved	Translation hypothetical protein	223.8
EHI_126560	AIG1 family protein, putative	Stress response	70.9
EHI_012330	Serine-threonine-isoleucine rich protein, putative	Miscellaneous	61.1
EHI_025700	Serine-threonine-isoleucine rich protein, putative	Miscellaneous	59.5
EHI_144280	Hypothetical protein	Hypothetical protein	48.8
EHI_144490	Hypothetical protein, conserved	Stress response hypothetical protein	38.9
EHI_126550	AIG1 family protein, putative	Stress response	36.0
EHI_082070	Rab family GTPase	Membrane traffic	31.2
EHI_004340	Serine-threonine-isoleucine rich protein, putative	Miscellaneous	31.0
EHI_176800	Zinc finger protein, putative	Transcription	27.6

Data collected from Transcript Expression Array Profiles in Amoeba DB (http://amoebadb.org/amoeba/).

Table S3. Top ten downregulated genes in strain G3 compared with strain HM-1:IMSS.

Gene ID	Product Description	Cat.	Fold Change (G3/HM-1:IMSS)
EHI_159480	Pore-forming peptide ameobapore A* precursor, putative	Miscellaneous	-490.5
EHI_194540	Pore-forming peptide ameobapore B* precursor, putative	Miscellaneous	-90.9
EHI_183680	Heat shock protein 101, putative	Chaperon	-21.2
EHI_155060	Chaperone clpB, putative	Chaperon	-20.6
EHI_094680	Chaperone clpB, putative	Chaperon	-20.1
EHI_090840	Chaperone clpB, putative	Chaperon	-19.8
EHI_070680	Hypothetical protein	Hypothetical protein	-18.6
EHI_022620	Heat shock protein, putative	Chaperon	-17.4
EHI_034710	Heat shock protein, putative	Chaperon	-17.4
EHI_017350	Heat shock protein, putative	Chaperon	-17.3

Data collected from Transcript Expression Array Profiles in AmoebaDB (http://amoebadb.org/amoeba/).

*G3 was developed as a strain that lacks the important virulence factor, amoebapore protein[1, 2].

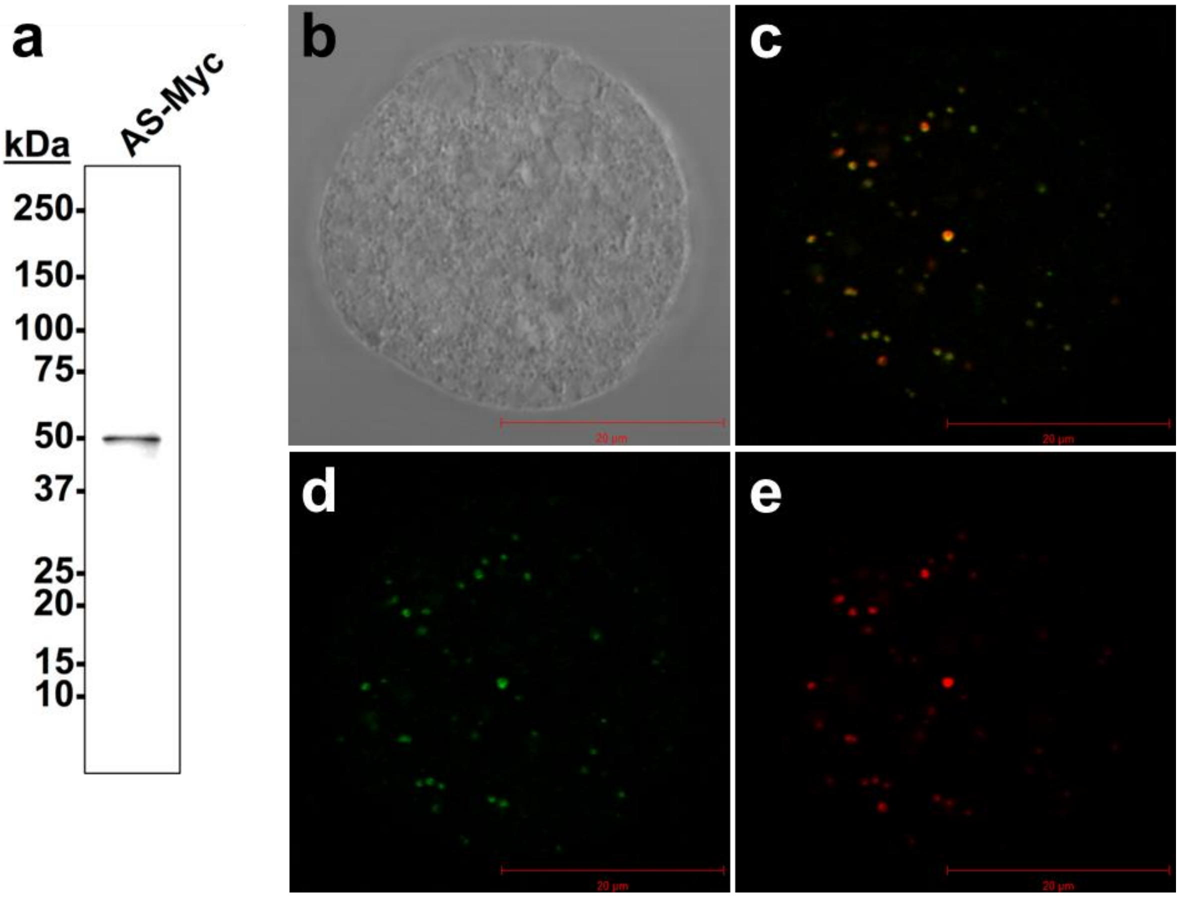

Fig. S1 | Expression of AS-Myc in *E. histolytica* HM-1:IMSS clone 6. a, Detection of AS-Myc by Western blotting. Whole cell lysate (20 µg protein) in SDS-PAGE sample buffer was boiled at 95°C for 3 min and then subjected to SDS-PAGE and immunoblotting[3]. Anti-Myc antibody (1:1,000 dilution, c-myc (9E10) monoclonal antibody, Covance) and anti-mouse immunoglobulin F(ab')2 fragment conjugated with horseradish peroxidase (1:3,000 dilution, Amersham) were used as the primary and secondary antibodies, respectively. **b-e,** Trophozoites expressing AS-Myc were stained by indirect immunofluorescence. Anti-Myc antibody (mouse) and anti-APSK antisera (rabbit) were used as primary antibodies (1:500 dilution). Alexa Fluor 594-labeled anti-mouse IgG antibody and Alexa Fluor 488-labeled anti-rabbit IgG antibody were used as secondary antibodies (1:400 and 1:2000 dilutions, respectively). Images of DIC, merge, Alexa Fluor 594 signals and Alexa Fluor 488 signals are shown in b, c, d and e, respectively. Bar indicates 20 µm.

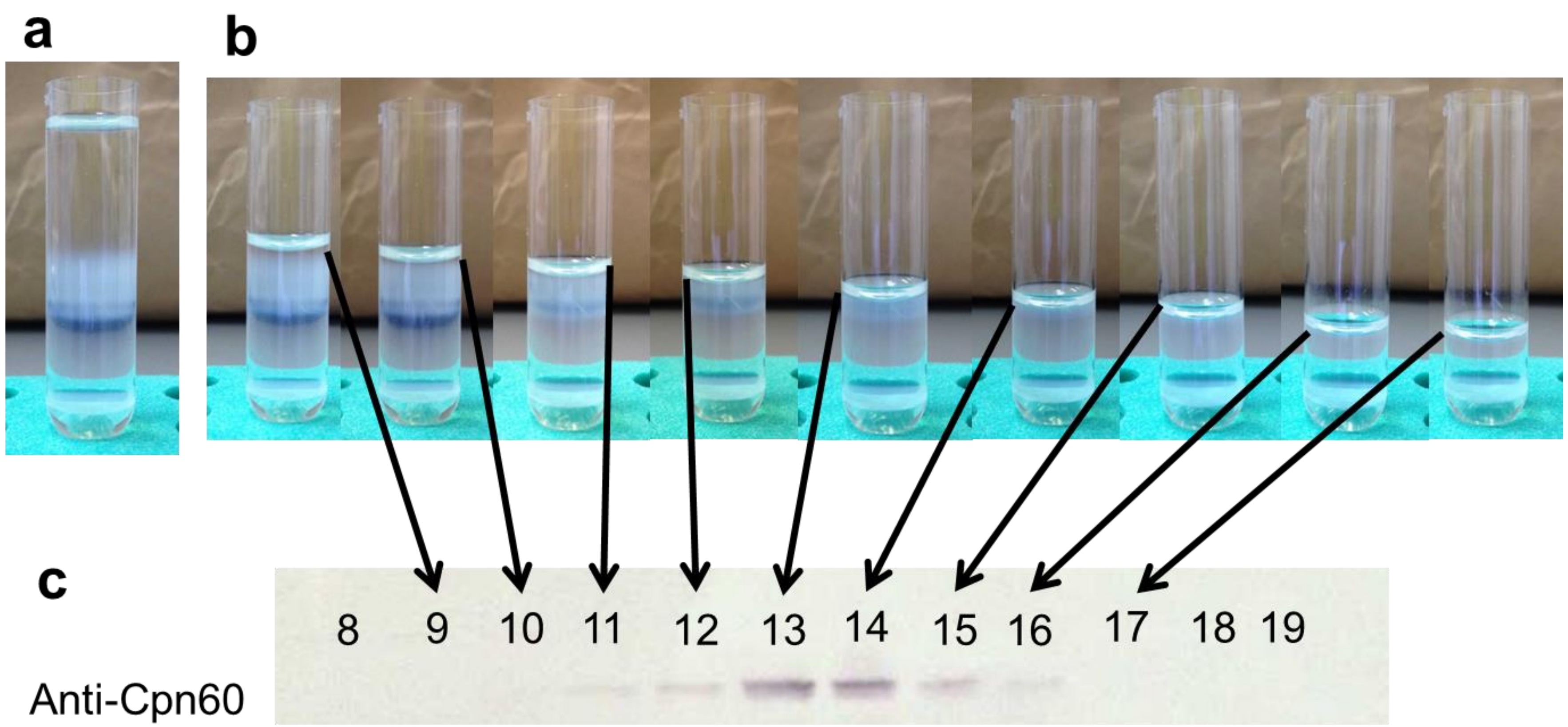

Fig. S2 | Western blotting analysis with rabbit anti-sera to Cpn60 for each fraction. a, Tube just after two-step Percoll-PLUS-gradient ultracentrifugation. **b**, Fractions (200 µl each) were collected from the top to the bottom of the tube. **c**, Mixtures of 2.5 µl of each fraction and 10 µl SDS-PAGE sample buffer were subjected to SDS-PAGE and immunoblotting. Anti-Cpn60 sera (1:1,000 dilution) and anti-rabbit immunoglobulin F(ab')2 fragment conjugated with alkaline phosphatase (1:2,000 dilution) were used as the primary and secondary antibodies, respectively. The numbers in c corresponds to the fraction number.

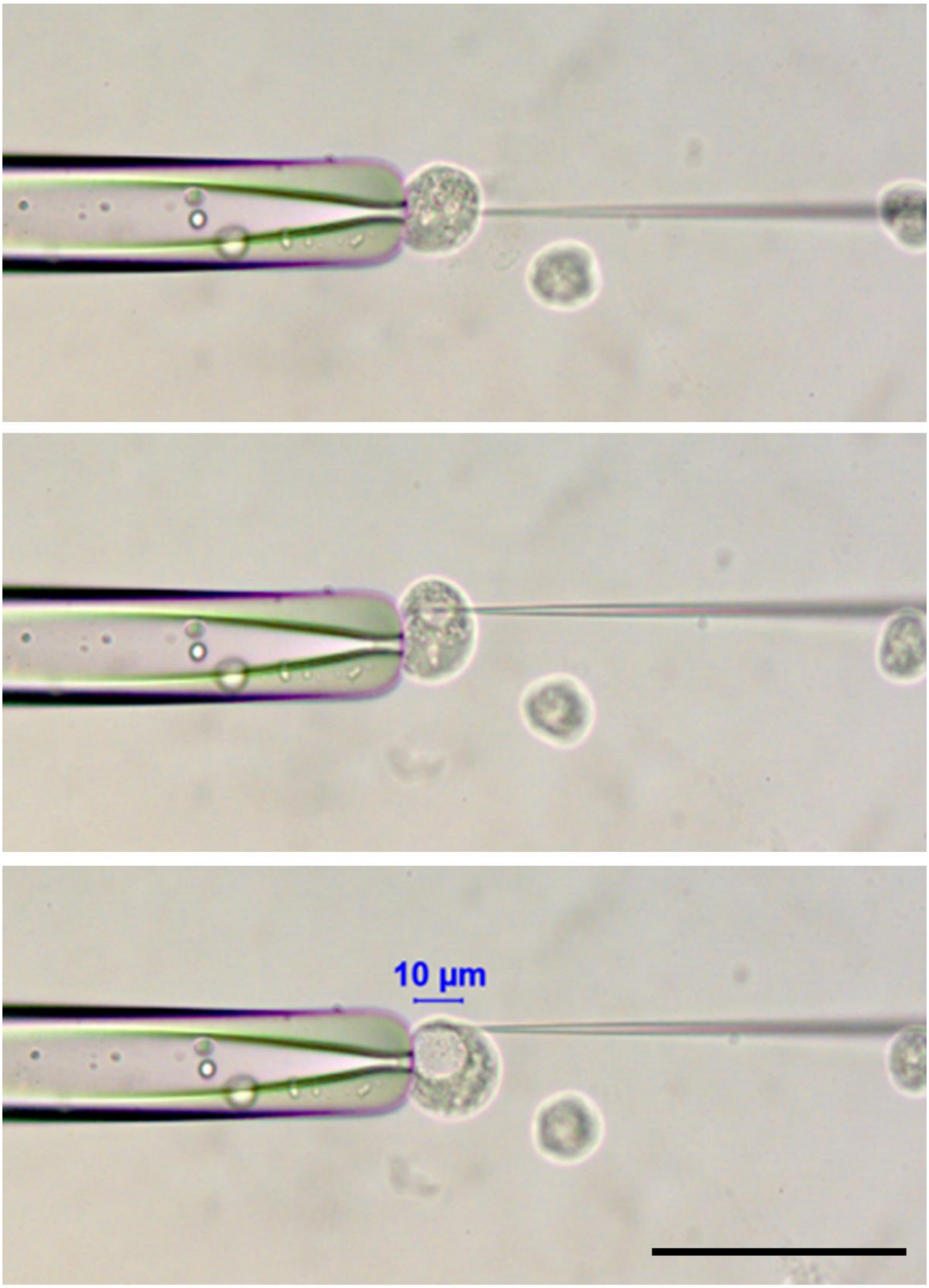

Fig. S3 | Three continuous images during microinjection into trophozoites of *Entamoeba invadens*. This recipient cell showed a clear space indicating a vacuole in the cytosol just after microinjection at 10°C. Such a vacuole-like structure was not observed in recipients of *E. histolytica*. The injection volume was estimated based on the diameter of the vacuole in cells measured just after injection. Bar: 50 µm.

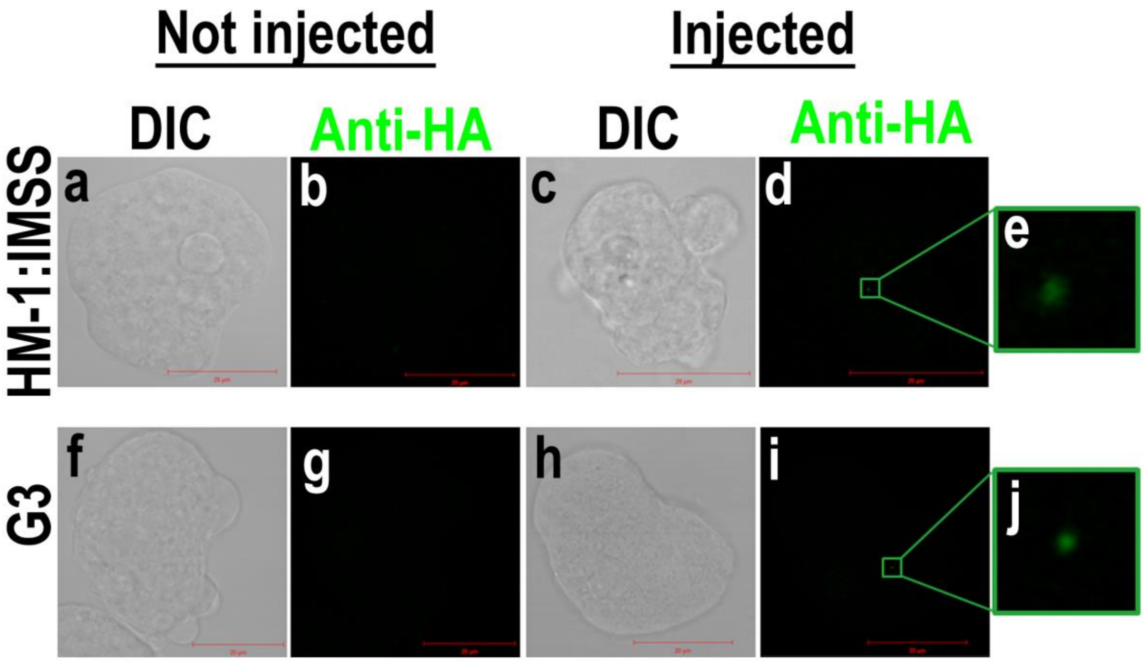

Fig. S4 | Donor mitosomes in recipient cells. *E. histolytica* strains HM-1: IMSS (a-e) and G3 (f-j) were used as recipients. Mitosomes were detected by immunofluorescent staining. a, c, f and h showed DIC images of trophozoites. b, d, g and i show fluorescent images. Magnified images of boxes in d and i are shown in e and j, respectively. Bar indicates 20 μm.

Video S1 | Time-lapse image of microinjection. Mitosomes containing APSK-HA were injected into a trophozoite expressing AS-Myc. The holding pipette and injection capillary are displayed on the left and right sides of the image, respectively. Different steps in this microinjection procedure are shown in Fig. 1g-i. Injected cells were placed on the limited peripheral area in the medium (Fig. 1l).

Video S2 | Cell divisions after microinjection. A trophozoite expressing AS-Myc after injection with mitosomes containing APSK-HA was transferred in new glass-bottom dish filled with fresh medium. After sealing, they were incubated at 37°C. This recording started from 40 min after microinjection. The trophozoite after injection divided in a binary manner at 10 h. Daughter cells showed binary fission at 29 h and 34 h.

Video S3 | Three-dimensional reconstruction of an injected cell. This is an animation of the cell shown in Fig. 3a and b by reconstruction of images. Red and green colors indicate AS-Myc and APSK-HA signals, respectively. Cytosolic fluorescence emissions are shown in white to make the cell shape clear. The dimensions of x and y indicates 55.7 µm.

References

1. Bujanover, S., Katz, U., Bracha, R. & Mirelman, D. A virulence attenuated amoebapore-less mutant of Entamoeba histolytica and its interaction with host cells. *Int. J. Parasitol.* **33**, 1655-1663 (2003).

2. Bracha, R., Nuchamowitz, Y. & Mirelman, D. Transcriptional silencing of an amoebapore gene in Entamoeba histolytica: molecular analysis and effect on pathogenicity. *Eukaryot. Cell.* **2**, 295-305 (2003).

3. Nakada-Tsukui, K., Saito-Nakano, Y., Ali, V. & Nozaki, T. A retromerlike complex is a novel Rab7 effector that is involved in the transport of the virulence factor cysteine protease in the enteric protozoan parasite *Entamoeba histolytica*. *Mol. Biol. Cell* **16**, 5294-5303 (2005).
